# Natural Silicates Encapsulated Enzymes as Green Biocatalysts for Degradation of Pharmaceuticals

**DOI:** 10.1021/acsestwater.3c00811

**Published:** 2024-01-30

**Authors:** Ani Vardanyan, Tatiana Agback, Oksana Golovko, Quentin Diétre, Gulaim A. Seisenbaeva

**Affiliations:** † Department of Molecular Sciences, Swedish University of Agricultural Sciences, P.O. Box 7015, Uppsala 75007, Sweden; ‡ Department of Aquatic Sciences and Assessment, Swedish University of Agricultural Sciences, P.O. Box 7050, Uppsala 75007, Sweden

**Keywords:** sol−gel, enzyme catalysis, silicates, immobilization, enzymatic degradation

## Abstract

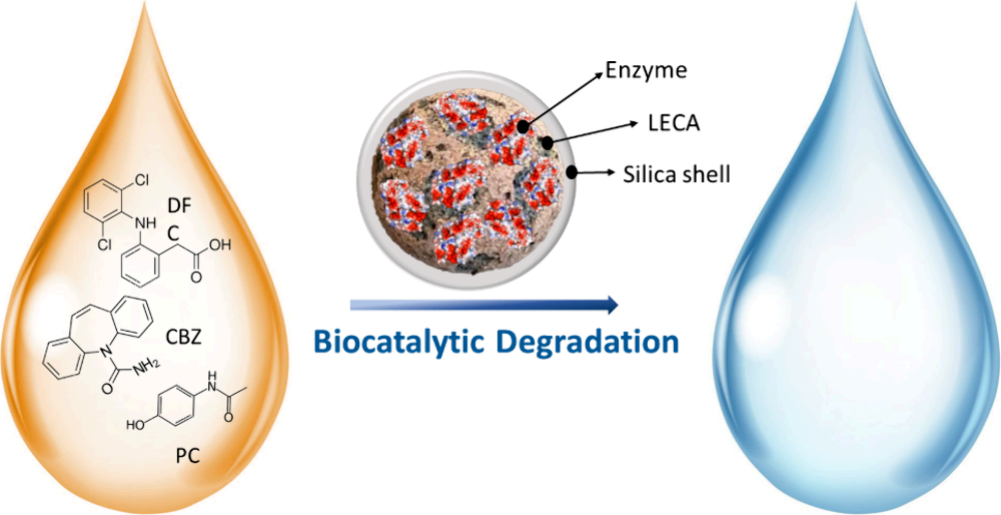

Biocatalytic degradation with the use of enzymes has gained great attention in the past few years due to its advantages of high efficiency and environmental friendliness. Novel, cost-effective, and green nanoadsorbents were produced in this study, using natural silicates as an enzyme host matrix for core–shell immobilization technique. With the natural silicate as a core and silica layer as a shell, it was possible to encapsulate two different enzymes: horseradish peroxidase (HRP) and laccase, for removal and degradation of three pharmaceuticals: diclofenac (DFC), carbamazepine (CBZ), and paracetamol (PC). The biocatalysts demonstrated high oxidation rates for the selected pollutants. In particular HRP immobilized fly ash and perlite degraded DFC and PC completely during 3 days of interaction and also showed high degradation rates for CBZ. Immobilized laccase was successful in PC degradation, where up to 70–80% degradation of the compounds with aromatic rings was reported by NMR measurements for a high drug concentration of 10 μg/mL. The immobilization method played a significant role in this process by providing stability and protection for the enzymes over 3 weeks. Furthermore, the enzymes acted differently in the three chosen supports due to their complex chemical composition, which could have an effect on the overall enzyme activity.

## Abstract 2

Core−shell immobilization of enzymes into natural silicates permitted appreciable degradation of pharmaceuticals. The immobilization method played significant role in this process by providing stability and protection for the enzymes.

## Introduction

During the past few decades, the production and consumption of pharmaceutical products have rapidly increased with the development of human and veterinary medicine.^1^ The presence of many pharmaceuticals have been reported in different water bodies all over the world.^2,3^ Although the long-term effects of these compounds are not yet well understood, studies showed that most representative pharmaceuticals, such as carbamazepine, diclofenac, oxazepam, paracetamol (acetaminophen), and others have ecotoxicological effects on aquatic organisms.^4^

Wastewater and drinking water treatment plants (WWTP) are not normally designed to remove pharmaceutical residues and other organic pollutants that are difficult to break down. In fact, studies showed that WWTPs are the main sources of such pollutants that can pass through the system and enter water bodies.^5,6^ Alternative methods are being developed continuously to find more efficient, cost-effective, and environmentally friendly ways to remove or degrade these pollutants.

The transformation of harmful contaminants catalyzed by enzymes is considered a highly efficient and green way of water treatment. In particular, oxidoreductases like peroxidases and laccases are capable of oxidizing different phenolic compounds, polychlorinated biphenyls (PCBs), industrial dyes, polycyclic aromatic hydrocarbons (PAHs) and other xenobiotics.^7^ The major drawbacks, such as low stability and nonreusability, have been addressed in different studies, and potential strategies were suggested to overcome these limitations. As such, immobilization on different supports provides the possibility of enzyme reuse and prolonged stability under different operational conditions. Various substrates have been tested for a successful enzyme immobilization including natural silicates,^8^ mesoporous silica nanoparticles,^9−11^ nanofibers,^12^ etc. Previously, the successful immobilization and subsequent removal of different pharmaceuticals, such as acetaminophen and DFC in the presence of Cd (II), was performed by our group via cross-linking laccase on Fe_3_O_4_/SiO_2_-DTPA (diethylenetriaminepentaacetic acid) hybrid nanocomposites.^13^ Taheran et al. (2017) reported the use of laccase covalently immobilized onto nanofibrous membrane and tested for degradation of chlortetracycline (CTC), CBZ, and DFC.^14^ The batch experiments revealed 72.7%, 63.3%, and 48.6% degradation efficiency for DFC, CTC, and CBZ at ppb ranges of these contaminants. In another study, Zhang et al. (2010) used graphene oxide to immobilize HRP for removal of seven different phenolic compounds. For some of the tested phenolic pollutants, the removal efficiencies were above 69% (4-methoxyphenol, 2-methoxyphenol, 3-aminophenol), and for catechol, the number exceeded 80%, showing promising results for using these types of biocatalysts for water purification.^15^

An important aspect to consider for any enzymatic purification technology is the cost effectiveness of the immobilization process, including the choice of substrates. In general, enzyme immobilization can be divided into two main methods: physical and chemical. Physical enzyme immobilization through entrapment or encapsulation represents a cost-effective, easy to handle immobilization technique that does not require any structural modifications of the enzyme, which often leads to a decrease in activity. However, immobilization based on simple adsorption or entrapment often leads to enzyme leakage, a phenomenon that should be prevented in water treatment processes.^16,17^ Here, we immobilized laccase and HRP enzymes on three different natural substrates for the removal of model pharmaceutical compounds found in most WWTPs. The search for such cost-effective natural supports has oriented our study toward siliceous materials perlite, lightweight expanded clay aggregate (leca), and fly ash (FA). Perlite and leca are amorphous aluminosilicates that are widely used, especially for the plant growing industry. Being porous materials they can be promising supports for immobilization of different enzymes and their subsequent use for water purification.^18−21^ FA on the other hand, is an industrial byproduct derived from waste incineration and has a potential for use as a support in enzyme technology,^22−24^ as it both solves disposal problems and is economically cheap.

**Figure 1:**
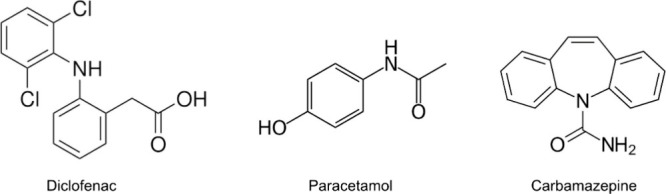
Chemical structure of DFC, PC, and CBZ.

To avoid enzymes’ leaching and activity loss, herein we report a new core–shell immobilization technique on natural silicates and their further investigation for removal and degradation of CBZ, DFC, and PC (Figure 1). Enzyme shielding was reported previously by Shahgaldian et al., (2016) where they produced hybrid organic–inorganic nanobiocatalysts by self-assembly of silane building blocks at the surface of enzymes which enhanced resistance of enzymes to denaturing stresses.^25^ To the best of our knowledge, the preparation of laccase and HRP immobilized natural silicates covered with a silica layer has not yet been reported. Thus, we present a combined approach consisting of the immobilization of these two enzymes through adsorption followed by encapsulation into a silica matrix, which we suppose will increase the enzymes’ operational stability and overall degradation efficiency. The immobilization process was properly optimized with reference to the immobilization yield and activity of the enzymes.

## Materials and Methods

### Materials

For the synthetic procedures, the following reagents have been used: tetraethoxysilane (TEOS), Sigma-Aldrich Sweden AB, CAS 8,00658, Stockholm, Sweden; sodium acetate trihydrate, Sigma-Aldrich, CAS 6131-90-4; ammonium fluoride, Sigma-Aldrich, Sweden AB, CAS 12125-01-8; acetic acid, Sigma-Aldrich, CAS 64-19-7; hydrogen peroxide, Sigma-Aldrich, CAS 7722-4-1; diclofenac sodium salt, Sigma-Aldrich, CAS 15307-79-6; acetaminophen, Sigma-Aldrich, CAS 103-90-2; carbamazepine, Sigma-Aldrich, CAS 298-46-4; 2,2′-azino-bis(3-ethylbenzothiazoline-6-sulfonic acid) diammonium salt (ABTS), Sigma-Aldrich, CAS 30931-67-0; leca, Blomster Landet, Sweden; perlite, Impecta Fröhandel, Sweden. Fly ash samples were obtained from Easy Mining company, Uppsala, Sweden. The enzymes were purchased from Sigma-Aldrich with their enzyme activity details specified on the bottles: HRP, CAS 9003-99-0, 156 U/mg; laccase (from Trametes versicolor), CAS 80498-15-3, 1.02 U/mg. The purity of laccase was checked by SDS-PAGE (sodium dodecyl sulfate–polyacrylamide gel electrophoresis) using 4–20% precast polyacrylamide gel with the standard ladder of Protean stain-free precision plus protein standards (Figure S15).

For the HPLC analysis, the reference standards were purchased from Sigma-Aldrich (Sweden). Isotopically labeled internal standards were purchased from Wellington Laboratories (Canada) and Toronto Research Chemicals (Toronto, Canada). All analytical standards were of high analytical grade (>95%).

### Methods

Particles were morphologically characterized by scanning electron microscopy using Hitachi (Tokyo, Japan) Flex-SEM 1000 environmental scanning electron microscope at an acceleration voltage of 5 kV, a spot size of 20, and a working distance of 5 mm. Elemental analysis of surfaces were performed using scanning electron microscopy with energy dispersion spectroscopy (EDS) (Hitachi (Tokyo, Japan) Flex-SEM 1000 environmental scanning electron microscope combined with AZtecOneXplore EDS detector by Oxford instruments (UK)). For each sample in EDS analyses, at least 5 different areas were studied, and an acceleration voltage of 20 kV, a spot size of 50, and a working distance of 10 mm were used. The average value was then calculated and given as the relative content of the elements.

The pore volumes were estimated by pycnometric measurements (Supporting Information).

Enzyme activity was measured by UV–vis spectroscopy at λ = 420 nm using a Multiskan Sky High (Thermo Fisher Scientific, Waltham, MA, USA) apparatus and standard 96 well plates.

Fourier-transform infrared (FTIR) spectra of the natural silicates before enzyme immobilization were recorded as KBr pellets using a demountable cell with KBr glasses on a PerkinElmer Spectrum 100 instrument.

Concentrations of drugs and degradation kinetics were obtained using NMR, HPLC and UV–vis spectroscopy. For UV–vis measurements, the absorption was recorded between 200 and 600 nm, and maximum absorption wavelength was determined accordingly (λ = 243 nm for PC, λ = 273 nm for DFC, and at λ = 285 nm for CBZ). All solutions were filtered through 0.2 μm cellulose membrane filters in order to separate the composite particles. Measurements were done on a Multiskan Sky High (Thermo Fisher Scientific, Waltham, MA, USA) apparatus with standard quartz cells.

### NMR Studies

The NMR experiments were acquired on Bruker Avance III spectrometers, operating at 14.1 T, that were equipped with a cryo enhanced QCI-P probe at a temperature of 298 K. Chemical shifts were referenced to TMS at 0.0 ppm. Data were processed and analyzed with TopSpin 4.3.0 (Bruker).

For all experiments after removal of the nanocomposite, the sample solution was filtered through 0.2 μm cellulose membranes. The final water solution, 500 μL, contained 10% of D_2_O.

### HPLC Analysis

One milliliter of the filtered sample was spiked with 10 ng of internal standards of DFC (13C6) (for quantification of DFC concentration) and CBZ (D10) (for quantification of CBZ concentration) per aliquot of sample. The samples were analyzed by a DIONEX UltiMate 3000 ultrahigh pressure liquid chromatography (UPLC) system (Thermo Scientific, Waltham, MA, USA) coupled to a triple quadrupole mass spectrometer (MS/MS) (TSQ QUANTIVA, Thermo SCIENTIFIC, Waltham, MA, USA). An Acquity UPLC BEH-C18 column (Waters, 100 mm × 2.1 i.d., 1.7 μm particle size from Waters Corporation, Manchester, UK) was used as an analytical column. The injection volume was 10 μL for all samples. A heated electrospray ionization (H-ESI) was used to ionize the target compound. The spray voltage was set to static: positive ion (V) 3500. Nitrogen (purity >99.999%) was used as a sheath gas (50 arbitrary units), auxiliary gas (15 arbitrary units), and sweep gas (2 arbitrary units). The vaporizer was heated to 400 °C and the capillary to 325 °C. The mobile phase consisted of Milli-Q water with 5 mM ammonium acetate and acetonitrile. The flow rate was 0.5 mL/min and run time was 15 min. Xcalibur software (Thermo Fisher Scientific, San Jose, CA, USA) was used for optimizing the instrument methods and running of samples. The obtained data were evaluated using TraceFinderTM 3.3. Software (Thermo Fisher). No target compounds were detected in method blanks and control samples.

### Immobilization of Enzymes by Their Encapsulation into Silica Matrix

300 mg of adsorbent material (leca, perlite, or FA) was suspended in 10 mL of enzyme solution with specific enzyme concentrations (100 U/mL for HRP and 20 U/mL for laccase) and let the enzyme to adsorb for 24 h. Afterward 25 mL of ethanol, 15 mL of water, and 0.2 mL of 1% NH_4_F in water was added. To get the silica shell, 4 mL of TEOS in 5 mL of ethanol (EtOH) was added dropwise over 30 min. After several hours, the solution became viscous and transformed into a gel. The mature gel was washed with water and ethanol three times each, and the composites were then freeze-dried overnight. To calculate the enzyme loading, the water and ethanol solutions were collected after each washing, and the enzyme concentration was measured by Bradford and enzyme activity assays. For Bradford assay, two calibration curves were established using bovine serum albumin (BSA) protein standards in water with final concentrations ranging between 0.1 and 1.4 mg/mL and 1–8 μg/mL for lower concentrations of enzymes (Figure S1). For the standards and the unknown enzyme sample (HRP or laccase) with a volume of 0.1 mL, 3 mL of Bradford reagent was added and left to react for 30 min. The samples were then transferred into cuvettes and measured for their absorbance at 595 nm by UV–vis. By this assay, it was possible to calculate the amount of enzyme that was loaded on core–shell biocatalysts. However, some enzyme can lose their activity during the immobilization, and to be able to estimate the active enzyme amount, we measured the enzyme activity before (initial enzyme solution with 100 and 20 U/mL concentrations) and after immobilization (in the supernatant after separating the biocatalysts) and the loading was calculated as the enzyme activity difference.

### ABTS Oxidation Test for Determination of Free Enzymes Activity

HRP and laccase enzymes can oxidize ABTS dye which turns from transparent (light green) to dark green color, and this color change can be monitored by UV–vis spectrometry. Enzyme activity was determined by monitoring the rate of oxidation of ABTS dye at 420 nm. One unit of enzyme activity was defined as the amount of enzyme required to oxidize 1 μmol of ABTS (molar extinction coefficient ε_420_ = 36 000 M^–1^ cm^–1^)^26^ per minute per unit volume and is expressed in U/mL. Triplicate measurements were performed for each assay of enzyme activity. ABTS was prepared in a potassium phosphate buffer (pH 6.5, 0.1 M) and a final concentration of 0.2 mM. The assay was performed in 96 well plate, where the solution in each well cell contained 90 μL of ABTS and 10 μL of 0.2 U/mL enzyme solution. For activity assay with peroxidase enzyme, 30 μL of 3.6% (1.2 M) hydrogen peroxide was added to 1 mL of ABTS stock solution to activate the enzyme.

### ABTS Oxidation Test for Determination of Immobilized Enzymes Activity

To determine the activity of core–shell immobilized enzymes, 100 mg of biocatalyst samples was placed in 10 mL of ABTS solution (0.2 mM) which started to change its color from transparent to green. In the case with the HRP enzyme, 30 μL of 3.6% hydrogen peroxide was added to the ABTS solution to start the reaction. To follow ABTS color change, 100 μL aliquots of reaction solution were taken every set time of intervals, filtered with syringe filters, and measured for their absorption at 420 nm by UV–vis spectrometry. The activity of the enzyme was then determined by calculating the rate of ABTS oxidation by its color change. It is important to note that the enzyme activity should be calculated before the enzyme kinetic curve reaches its saturation, which is why it is advised to collect at least 3 samples from the reaction mixture, to check the linearity of the enzyme activity graph.

### Immobilized Enzyme Stability and Reusability

To determine a better storage temperature for the immobilized enzymes, the samples were divided into two parts and kept in the fridge or room temperature. For 3 weeks, every day, biocatalyst samples (core–shell immobilized HRP and laccase) were tested by the ABTS oxidation assay described earlier, with slight modifications. Briefly, 10 mg of sample (core–shell immobilized enzyme) was mixed with 10 mL of ABTS solution (0.2 mM) and the color change of ABTS was monitored by UV–vis absorption measurements at 420 nm. Relative enzyme activity (*A*_R_) was then calculated according to this equation:
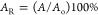
Where *A* is the enzyme activity measured every day, and *A*_o_ is the activity measured on the first day of the experiment.

For reusability experiment, 100 mg of biocatalyst samples was mixed with 10 mL of ABTS solution and the activity was calculated by the method described earlier. After the first cycle of activity measurement, ABTS was removed by centrifugation (7000 g), and 10 mL of phosphate buffer (pH 6.5) was added to the samples (core–shell immobilized enzymes) and put on a shaker overnight to get rid of access ABTS. The following cycles were done with a fresh ABTS solution and the same experimental conditions. For both stability and reusability experiments, samples were measured in triplicates and standard deviations were calculated.

### Drugs Degradation by Immobilized Core–Shell Enzymes

DFC, CBZ, and PC solutions were prepared in water with different initial concentrations ranging from 3 to 20 μg/mL. DFC was tested at two different concentrations, 3 and 10 μg/mL, PC was tested at 10 and 20 μg/mLand CBZ was tested at an initial concentration of 20 μg/mL. 100 mg of natural silicates with immobilized enzymes was mixed with 10 mL of drug solution and put on a shaker for 24 h at room temperature. After 24 h, biocatalysts were separated from the solution, and the supernatant was tested for residual pharmaceuticals. Control samples (natural silicates) without immobilized enzymes were tested for drug removal via adsorption in the same conditions, and the concentrations of the drugs were measured by UV–vis and HPLC methods. For kinetic experiments, core–shell particles with enzymes were mixed with drug solution, and samples were taken in different time intervals. All samples were filtered (syringe filter, 0.22 μm) before analytical measurements.

## Results and Discussion

### Immobilization of Enzymes and Their Encapsulation into Silica Matrix

In the first step of enzyme immobilization, we used three different supports to encapsulate the HRP and laccase enzymes. To obtain core–shell structure with adsorbed enzyme core and a membrane that is permeable for the substrates of the enzymes, Sol–Gel chemistry was applied^27^ where TEOS was chosen as a silica source and ammonium fluoride as a catalyst. In order to keep the activity of immobilized biomolecules for longer periods, the concentration of catalyst and the volume of the solvent (ethanol) was optimized and kept to a minimum. The hydrolysis of the precursor and its subsequent condensation resulted in a silica shell that covered the support with the entrapped enzyme.

To confirm the first and second steps of enzyme immobilization, the concentration and activity of enzyme was measured before and after immobilization. Further, the silica shell formation was monitored by EDS measurements, which showed an increase in silica content in adsorbents surfaces from 10% to 23% (atomic weight) (Table S1 and S2, Figure S2 and S3 in the Supporting Information).

**Figure 2:**
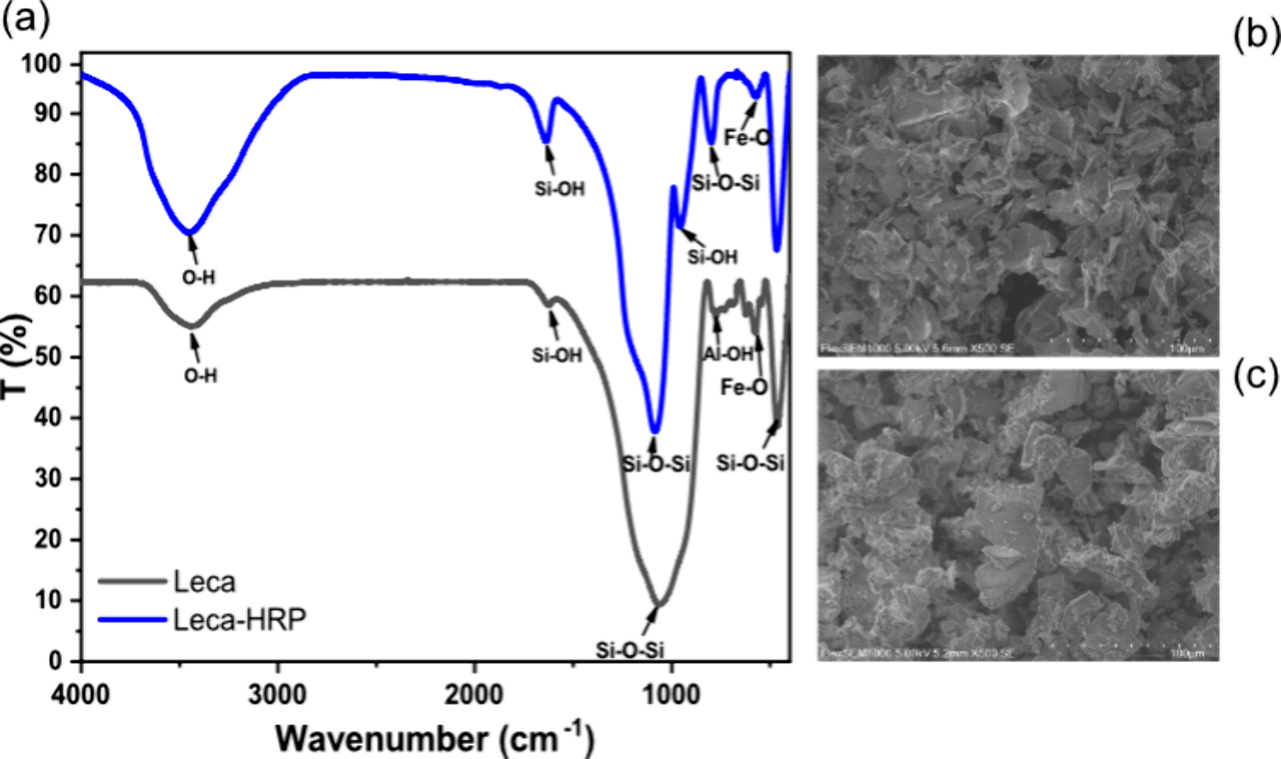
(a) FTIR spectra of leca and HRP immobilized core–shell leca samples; SEM images of perlite (b) before and (c) after silica layer formation.

Furthermore, SEM images of natural adsorbents (Figure 2 b, c and Figure S4) before and after silica shell formation demonstrated some morphological differences. Figure 2b shows the flake like particles of perlite before deposition of a silica cover and more bulky and aggregated particles after shell formation (Figure 2c).

Appearance of the silica shell was additionally monitored by FTIR analysis on bare adsorbents and core–shell immobilized samples. All three samples had characteristic peaks of SiO_2_ around 460, 800, and 1085 cm^–1^, corresponding to δ(Si–O–Si), υ(Si–O–Si), and υ_as_(Si–O–Si) vibrations^28,29^ (Figure 2a, Figure S5). Another characteristic band for leca and FA samples (Figure S5) appeared at 572 cm^–1^ and a shoulder peak at 730 cm^–1^ that could correspond to Fe–O and Al–OH groups.^30−32^ The latter disappeared after silica shell coverage and instead a new band appeared for all three samples at 960 cm^–1^ that corresponded to Si–OH vibrations.^33^

**Figure 3:**
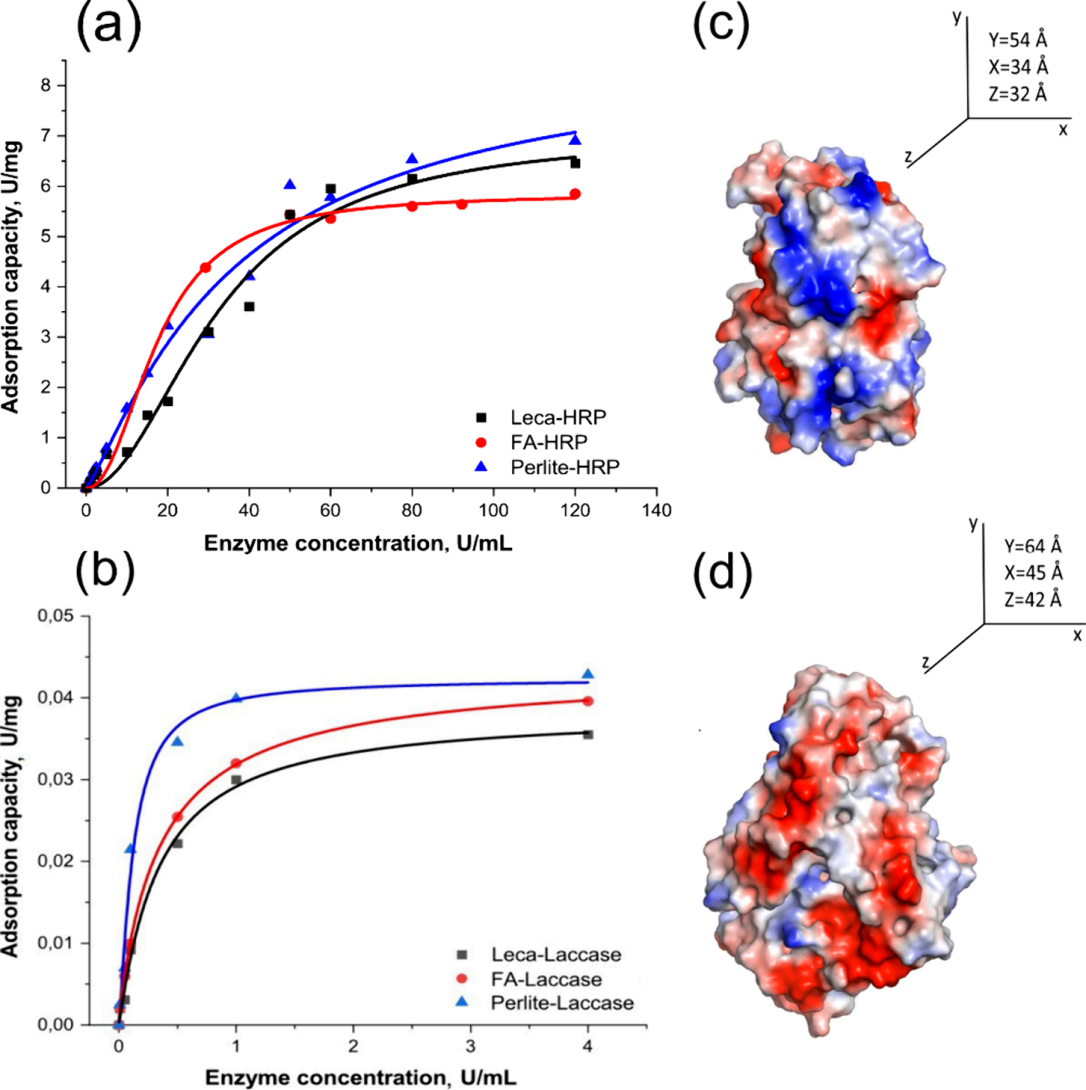
Adsorption of (a) HRP and (b) laccase on natural silicates at different initial enzyme concentrations obtained by enzyme activity assay; enzyme dimensions (PyMol software) for (c) HRP and (d) laccase. Red color represents negatively charged amino acid residues while blue ones are positively charged residues.

In order to confirm and calculate the enzyme loading, Bradford assay and enzyme activity tests were performed before and after enzyme adsorption (Figure 3a, b). Both experiments confirmed the immobilization of the enzymes. The Bradford assay showed slightly higher enzyme loading compared to the enzyme activity assay (Table S3). This difference could mean that some enzymes lost their activity during the immobilization process. It has been previously reported that the immobilization of proteins on solid substrates may lead to secondary structural changes, with the extent depending on the amount of enzymes being adsorbed.^34^ In other words, as the amount of adsorbed enzymes increases, they become less stable due to an abundance of interprotein interactions that are unfavorable for their structural stability.^34^ This phenomenon could explain why some of the enzymes were deactivated during the immobilization process, which is why the enzyme activity test was chosen as the method for calculating the amount of the “live” enzymes loaded on the substrates.

Enzyme activity was also measured after the formation of a silica shell around the particles and gel formation. Compared to the amount of adsorbed enzyme, the core–shell immobilized enzyme showed slightly higher loading, probably due to additional entrapment under the silica shell. For HRP enzyme, the immobilization yield was 7.02, 7.5, and 6.4 U/mg (45, 48, and 41 mg/g) for leca, perlite, and FA respectively. For laccase the numbers were lower, 0.038, 0.041, and 0.043 U/mg (39; 42 and 44 mg/g) for leca, FA and perlite, respectively. The lower adsorption and consequent immobilization yield could be explained by the bigger enzyme size in case of laccase (Figure 3). Moreover, at neutral pH all three natural silicates demonstrate negative surface charge (Figure S6) while HRP enzyme has net positive surface charge and laccase has net negative charge (Figure 3c, d).^35−39^ Electrostatic interactions at different pH were shown to play a major role in the adsorption or interaction of enzymes with different surfaces.^40,41^

Comparing the adsorption rate of the enzymes on different natural silicates, we also noticed that perlite had higher adsorption capacity than leca and FA. This trend agreed well with the results from estimated pore volume for these three samples (Supporting Information). Compared to perlite, leca and FA had higher densities and lower pore volumes, approximately 0.32 cm^3^/g and 0.4 cm^3^/g, respectively, while for perlite, that number was almost twice as high, reaching 0.7 cm^3^/g. However, no noticeable correlation was found when we compared enzyme adsorption capacities between leca and FA. In general, the adsorption of enzymes onto different surfaces or porous substrates is quite complex and can be influenced by various factors like surface charge (both for enzyme and the substrate), hydrophobicity/hydrophilicity of the surfaces, pore size and structure, ionic strength, enzyme size and shape, enzyme specific interactions, etc.^42−47^ Further investigations are required to better understand how these factors affect enzyme adsorption onto natural silicates.

### Immobilized Enzyme Storage Stability and Reusability

**Figure 4:**
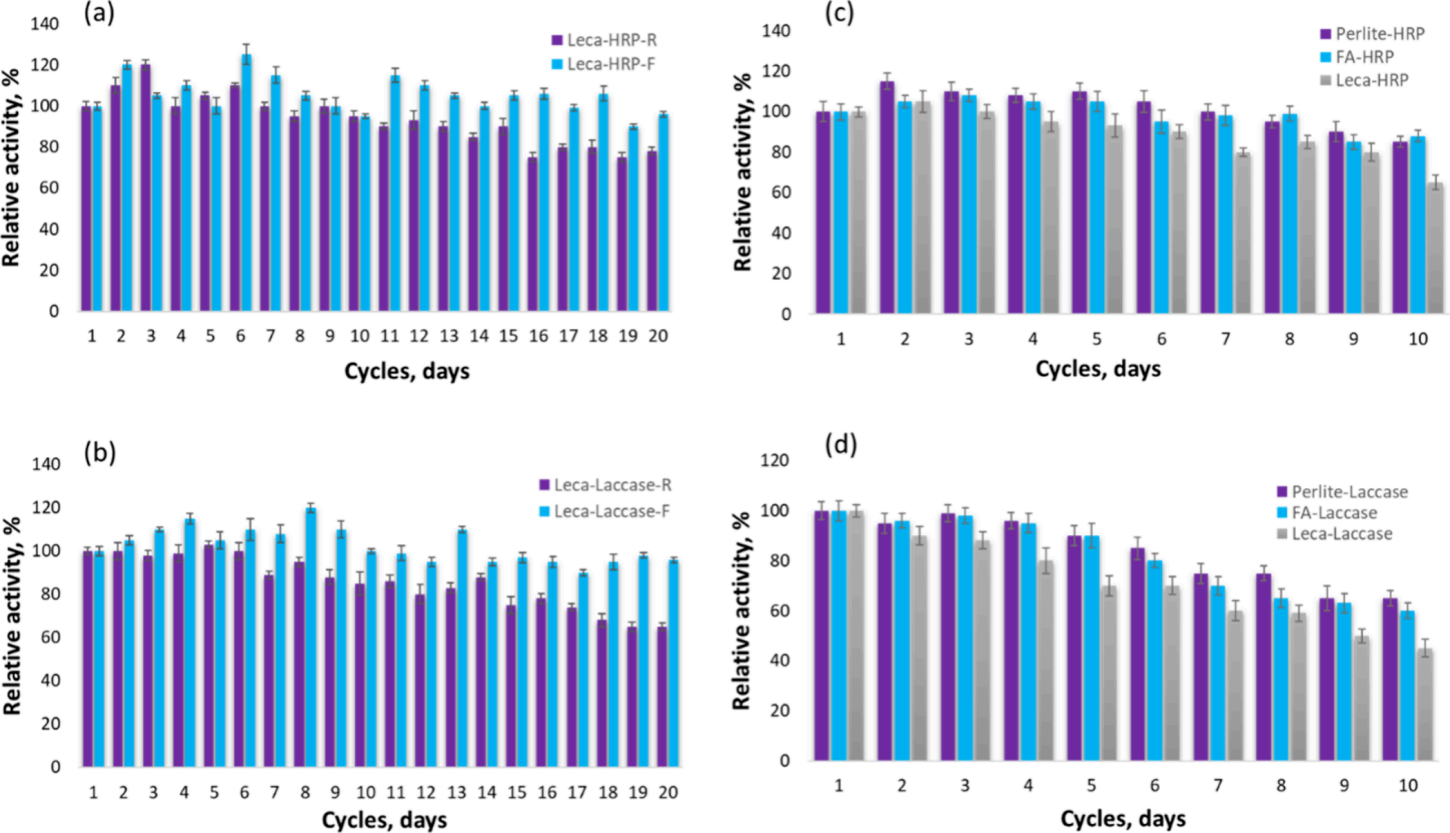
Stability of immobilized (a) HRP and (b) laccase on core–shell leca particles at room (R) and refrigerator (F) temperatures. Reusability of immobilized (c) HRP and (d) laccase into FA, leca, and perlite.

Enzymes can experience a decline in their catalytic effectiveness when stored for extended periods. Immobilization is one way to avoid this phenomenon by restricting the enzymes from leaching and protecting them from different unfavorable environmental parameters.^14^ Herein, the immobilized enzymes were tested for their storage stability and reusability in a ABTS oxidation test. For storage stability, the immobilized enzymes were kept at 4 and room temperature (25 °C). Comparing the two enzymes, we can notice that immobilized HRP was slightly more stable than laccase at room temperature. Both immobilized enzymes kept their activity very high (90–100%) in the fridge even after few weeks (Figure 4a, b, Figure S7). Additionally, it was noted that the enzymes immobilized in FA and perlite were more stable both at room temperature and in the fridge compared to the enzymes immobilized in leca (Figure 4a, b, Figure S7). These results were compared to free enzymes activity stored in potassium phosphate buffer (pH = 6.5) at room temperature and at 4 °C, with initial enzyme concentrations of 100 U/mL for HRP and 20 U/ml for Laccase. Figure S8 shows that both enzymes kept their activity for 3 weeks at 4 °C; however, laccase lost more than 50% activity after 5 days kept at room temperature. The results indicated that laccase immobilized on natural silicates can enhance its storage stability compared to the free enzyme molecules.

For the reusability test, the immobilized enzymes were mixed with ABTS and the activity was measured with UV–vis spectroscopy. After the first cycle, the ABTS was washed, and the experiment was continued with fresh ABTS solution. The results depicted in Figure 4c and d show that both enzymes could be reused for several cycles without losing their activity. Immobilized HRP showed better operational stability compared to laccase, keeping the relative enzyme activity above 80% after more than 6 cycles. Samples with leca started losing their partial activity after 3 cycles, keeping it above 50% for another 3 cycles and eventually dropping below 30%. Immobilized perlite and FA kept the enzyme (both HRP and laccase) activity for longer periods, and it started to drop after 5 cycles. The decrease in enzymes activity upon repeated usage could be connected with partial denaturation of the protein during the operation processes rather than enzyme leakage from the silica shell.^27,35^

### Drug Degradation by Immobilized Enzyme

**Figure 5:**
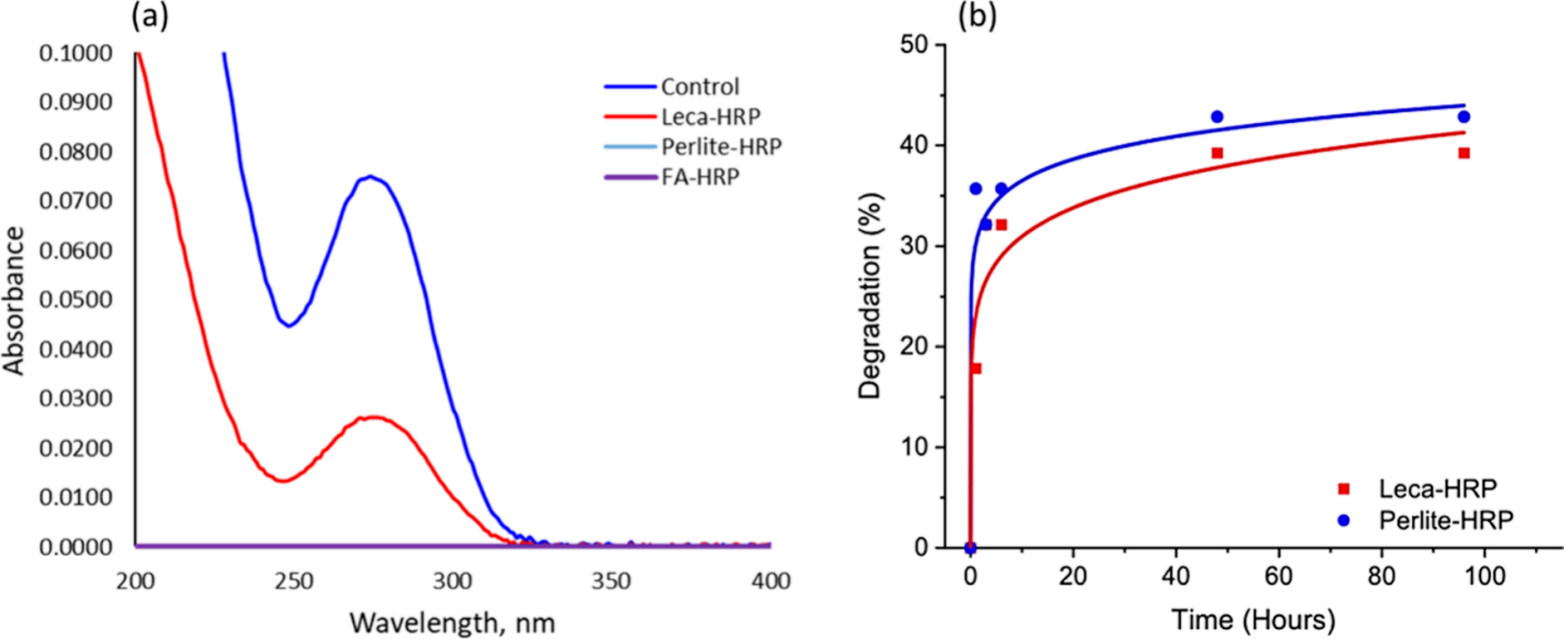
DFC degradation by immobilized HRP followed by UV–vis measurements and HPLC: (a) UV–vis measurements after 3 days of interaction, with an initial drug concentration of 3 μg/mL. (b) HPLC results of DFC degradation kinetics with an initial drug concentration of 10 μg/mL.

The removal of DFC, CBZ, and PC was investigated in a batch experiment, where the biocatalysts were suspended in the drug feed solution with known initial concentrations. In addition, control samples without the immobilized enzymes were tested for their capability for drug adsorption. The reactions were carried out at room temperature and pH 6.5. It is known that DFC is a recalcitrant drug due to its chemical structure with a chlorinated benzene ring. Low degradation rates (20–40%) of this drug in WWTPs have been reported previously.^48^ That is why it was exciting to notice almost complete degradation of DFC (at a concentration of 3 μg/mL) after interaction with our biocatalysts, specifically Perlite-HRP and FA-HRP. Since the adsorption experiments showed no noticeable drug removal by bare natural silicates, the results of biocatalytic removal were attributed to the degradation of pharmaceuticals, rather than their adsorption. NMR spectra for DFC at concentration of 3 μg/mL, before and after interaction with immobilized HRP are shown in Figure S9. The characteristic signals for the original drug were detected at 7.38 and 7.08 ppm, which can be attributed to 2,6-diclorophenyl ring protons. Phenylacetate ring protons resonate at 6.93 and 6.8 ppm.^49^ Most of the signals disappeared after interaction with perlite-HRP and FA-HRP samples, indicating almost complete degradation of DFC after 3 days. These results were confirmed by UV–vis measurements where the characteristic band for DFC decreased in intensity which could mean that DCF is converting into lower molecular weight carboxylic acids^50^ (Figure 5a).

The results showed high degradation rates (or complete degradation) for the samples of Perlite-HRP and FA-HRP, and approximately 65% degradation for Leca-HRP at a concentration of 3 μg/mL. The low degradation observed in the leca samples could be attributed to lower enzyme stability compared to Perlite and FA samples (Figure 4a, b, Figure S7). However, further experiments are necessary to fully understand the various interactions and effects that the substrates may have on the enzymes.

To be able to follow DFC degradation kinetics, a higher concentration of the drug solution (10 μg/mL) was prepared and two samples were tried (Perlite-HRP, Leca-HRP). The remaining DFC concentrations were measured by HPLC (Figure 5b). The results revealed fast degradation for both samples, reaching equilibrium in less than an hour for Leca-HRP and 3 h for Perlite-HRP. At concentration of 10 μg/mL, 43 and 40% of the drug was degraded by Perlite-HRP and Leca-HRP samples, respectively.

DFC degradation was tested also with immobilized laccase samples, which showed much lower degradation rates compared to HRP. For 10 μg/mL as initial DFC concentration, the degradation rates were calculated as 10, 6 and 4% for Perlite-laccase, FA-laccase, and Leca-laccase, respectively. Degradation rate was followed by UV–vis measurements after mixing the biocatalysts with drug solution and leaving to interact for 24 h. One difference between immobilized enzymes was that laccase activity was much lower than that of HRP. The free enzyme powder had activity of 1.02 U/mg, this number for HRP being 150–156 U/mg (Sigma-Aldrich). This means that after immobilization, the biocatalysts would have around 39 U/mg activity with immobilized laccase samples and more than 7000 U/mg activity with immobilized HRP samples.

To test the biocatalysts degradation capacity on other pharmaceuticals, CBZ and PC were chosen as representatives of two different groups: first one is an antiepileptic drug whose occurrence in different water bodies were reported worldwide including in Sweden.^51−53^ Compared to DFC and CBZ, the PC, a common analgesic drug, has a simpler chemical structure, containing only one benzene ring making it easier to degrade.^54^ However, being one of the most frequently prescribed or purchased medicine in many countries, high doses of this drug and its conjugates are constantly found in aquatic environments and drinking water, which is why it is important to find more effective degradation or removal methods to reduce PC pollution.^54^

PC degradation by HRP immobilized silicates was followed by UV–vis and NMR measurements at a concentration of 20 μg/mL and room temperature. UV–vis measurements showed that fast degradation occurred in the first 5–10 min (Figure S10) and slow degradation continued for the rest of the contact time with biocatalysts. Upon extended degradation, the peak at 246 nm gradually decreased, and another band appeared to grow with λ_max_ at 320 nm. This phenomenon was observed in previous studies with PC degradation, and could be explained with red-shifted absorption of ρ band of the ring.^55^ NMR spectra of the initial PC and PC after interaction with immobilized HRP were obtained after 3 days of the degradation period. From Figure S11 it is evident that immobilized HRP degraded PC completely.

**Figure 6:**
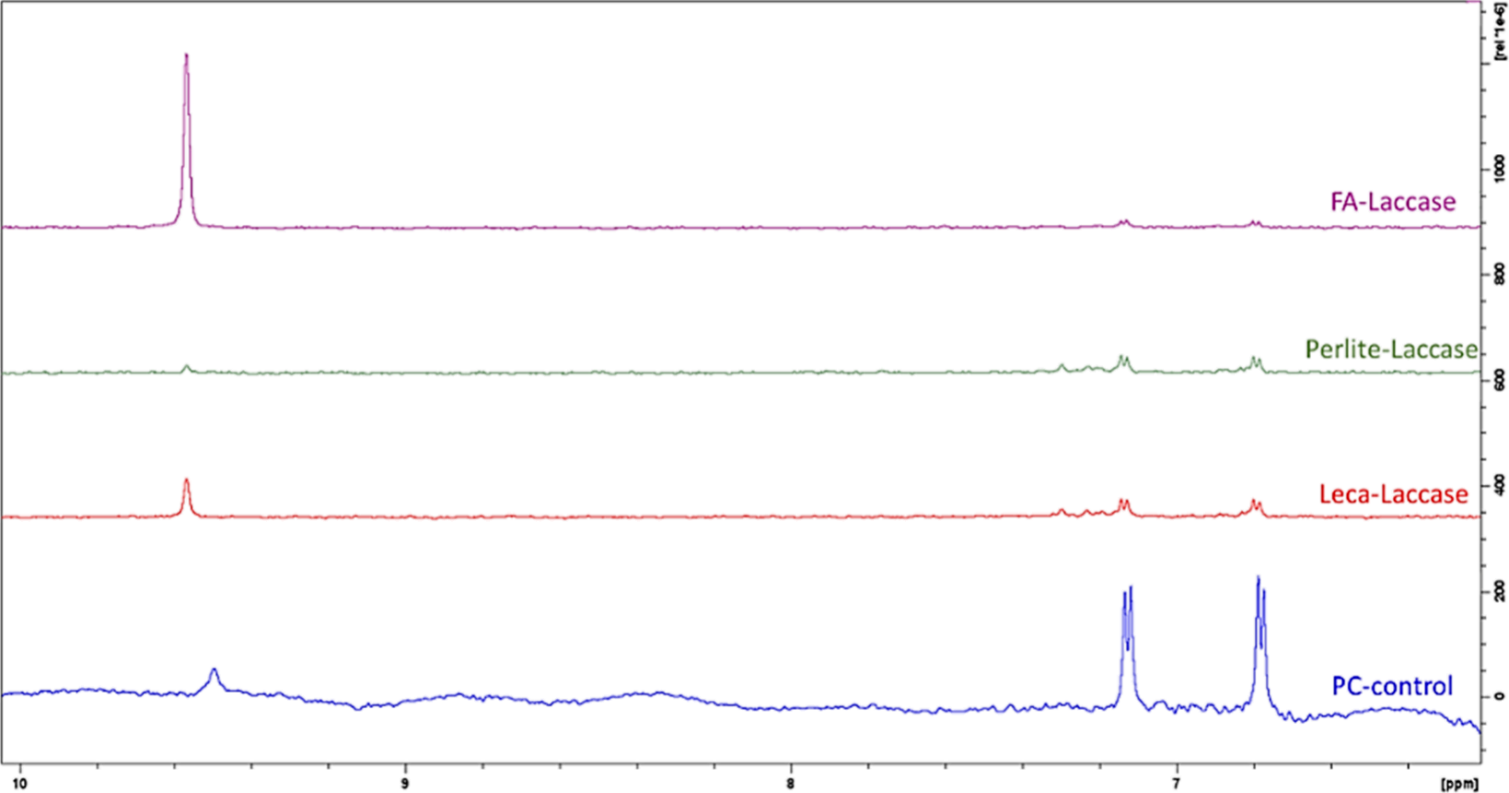
NMR spectra of initial PC (blue) and PC after 3 days of interaction with sol–gel encapsulated Perlite-laccase, FA-laccase, and Leca-laccase. The NMR sample solution was H_2_O:D_2_O 90%:10%.

PC degradation was also measured for immobilized laccase samples and showed lower degradation rates compared to HRP. Up to 10% PC degradation was achieved for initial PC concentration of 10 μg/mL in the first 10 min monitored by UV–vis (Figure S12). Slower degradation continued, which was confirmed by NMR measurements after 3 days of biocatalyst and PC interaction. Figure 6 shows that the aromatic ring had been cleaved by the enzyme, which led to the decrease in the peaks at 7.1 and 6.7 ppm. 70–80% degradation yield was achieved by immobilized Laccase.

According to UV–vis measurements, CBZ degradation (with initial concentration of 20 μg/mL) for immobilized laccase samples were calculated as 38%, 35% and 27% for Perlite, FA and leca samples, respectively. Approximately same numbers were calculated for immobilized HRP samples that were also obtained from UV–vis measurements: 35%, 32% and 23% for perlite, FA and leca, respectively (Figure S13). These results were also qualitatively confirmed by NMR measurements after 3 days of contact with biocatalysts, Figure S14. While CBZ is considered more recalcitrant and resistant to bio treatment or photodegradation, due to the presence of benzene rings (2 benzene rings connected with azepine ring)^56−58^ herein, we have reported a relatively good degradation capacity with our biocatalysts at very high drug concentration. These results showed that enzymatic degradation could perform well at concentrations that were several times higher than the concentrations found in WWTPs or natural water bodies.

## Conclusions

HRP and laccase enzymes were successfully immobilized on different natural silicates by two steps: adsorption and silica shell formation. The proposed method was advantageous compared to conventional covalent bonding, since most of the natural silicates that undergo heat treatment (expanded alumosilicates) lack active groups that could bind to the ligand (usually APTES and glutaraldehyde) and hence the enzyme. Adsorption is a simple way to immobilize different enzymes on a porous surface. Further formation of silica shell by sol–gel method helped preserve the enzymes inside the biocatalysts and protect it from the surrounding environment, which kept the enzyme activity longer compared to nonsilica shell adsorbents. Synthesized biocatalysts with immobilized HRP showed high degradation activity toward DFC and PC, removing both the drugs completely at initial drug concentrations of 10 and 20 μg/mL, respectively. CBZ degradation was also tested and revealed lower removal rates compared to DFC and PC, probably due to different oxidation pathway that was needed for this particular molecule. Additionally NMR experiments helped us to identify two intermediate products that are unstable and easier to degrade compared to the parent molecule. Higher concentrations of drugs ranging between 3 and 20 μg/mL were tested for all experiments compared to the actual concentrations found in the treatment plants for drinking water. The obtained results illustrated that the proposed biocatalysts can work effectively for the removal of different pharmaceuticals at low concentrations.

## Supporting Information Available

Calibration curves for Bradford assay, EDS mapping of leca before and after silica shell formation, leca samples before and after silica shell formation, FTIR spectra of bare perlite and FA compared to core–shell immobilized perlite and FA, the difference of enzyme loading measured by Bradford assay and enzyme activity test, zeta potential of natural silicates at natural pH, immobilized HRP and laccase storage stability at room (R) and 4 °C temperature (F), Storage stability of free enzymes at room (R) and 4 °C temperature (F), NMR spectra of DFC before and after interaction with immobilized HRP, Paracetamol degradation kinetics by Leca-HRP, FA-HRP, and Perlite-HRP samples, NMR spectra of initial Paracetamol (blue) and FParacetamol after interaction with sol–gel encapsulated Perlite-HRP, FA-HRP, and Leca-HRP, Paracetamol degradation by immobilized laccase after 10 min interaction with the biocatalysts, Carbamazepine degradation by immobilized HRP and laccase samples measured by UV–vis, NMR spectra of initial Carbamazepine (blue) and Carbamazepine after interaction with sol–gel encapsulated Perlite-laccase, FA-laccase, and Leca-laccase and HRP, and SDS-PAGE of Laccase enzyme (PDF)
