# Supplementary material for: Natural Silicates Encapsulated Enzymes as Green Biocatalysts for Degradation of Pharmaceuticals
Source: ACS ES T Water. 2024 Jan 30;4(2):751–60. doi: 10.1021/acsestwater.3c00811 (PMC10862536; doi:10.1021/acsestwater.3c00811)
Supplement: Supplementary file 1 — ew3c00811_si_001.pdf [file ew3c00811_si_001.pdf]

Supplementary materials

Natural Silicates Encapsulated Enzymes as Green Biocatalysts for Degradation of Pharmaceuticals

Ani Vardanyan[a]*, Tatiana Agback[a], Oksana Golovko[b], Quentin Diétre[a], Gulaim A. Seisenbaeva[a]*

a Department of Molecular Sciences, Swedish University of Agricultural Sciences, P.O. Box 7015, 75007 Uppsala, Sweden

b Department of Aquatic Sciences and Assessment, Swedish University of Agricultural Sciences, P.O. Box 7050, 75007 Uppsala, Sweden

Keywords: sol-gel, enzyme catalysis, silicates, immobilization, enzymatic degradation

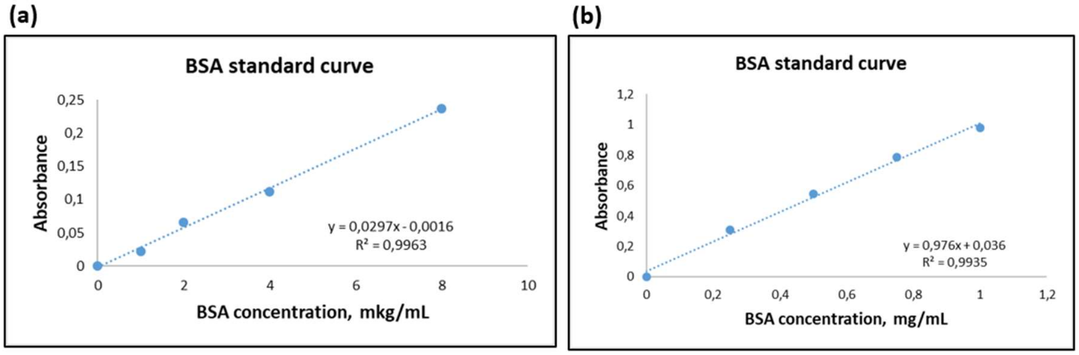

Figure S1. Calibration curves for Bradford assay at concentrations between 2-8 µg/mL (a) and 0.2-1 mg/mL (b).

Table S1. EDS analysis for leca before silica shell formation.

Map Sum Spectrum				
**Element**	**Line Type**	**Weight %**	**Weight % Sigma**	**Atomic %**
O	K series	42.90	0.86	50.26
Si	K series	15.33	0.33	10.23
Al	K series	7.16	0.18	4.97
Fe	K series	10.35	0.33	3.47
Mg	K series	1.36	0.08	1.05
K	K series	1.80	0.09	0.86
Ca	K series	2.26	0.10	1.06
Ti	K series	0.54	0.09	0.21
C	K series	17.47	1.50	27.27
S	K series	0.30	0.05	0.17
Na	K series	0.54	0.07	0.44
Total		100.00		100.00

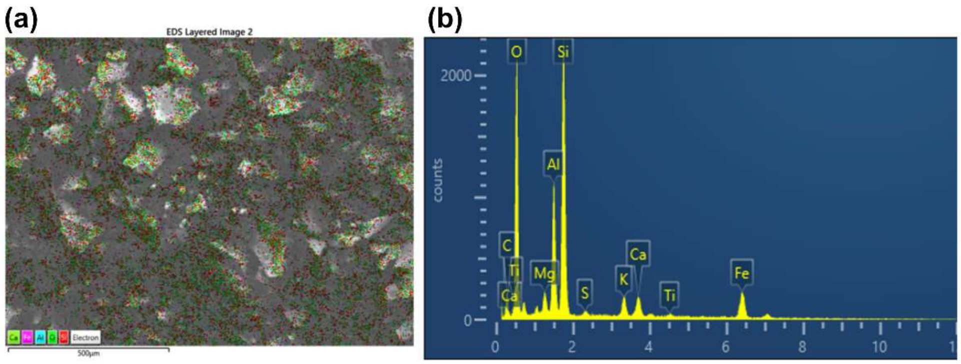

Figure S2. EDS layered image (a) and map spectrum (b) of leca sample before silica shell formation.

Table S2. EDS analysis for leca after silica shell formation.

Map Sum Spectrum				
**Element**	**Line Type**	**Weight %**	**Weight % Sigma**	**Atomic %**
Fe	K series	0.96	0.19	0.32
O	K series	52.62	2.04	60.51
Si	K series	35.10	1.37	22.99
K	K series	0.27	0.08	0.13
Al	K series	0.41	0.07	0.28
Na	K series	0.02	0.07	0.01
Mg	K series	0.04	0.06	0.03
Ca	K series	0.17	0.08	0.08

Ti	K series	0.04	0.09	0.01
C	K series	9.22	2.76	14.13
N	K series	1.15	2.23	1.52
S	K series	0.00	0.06	0.00
Total		100.00		100.00

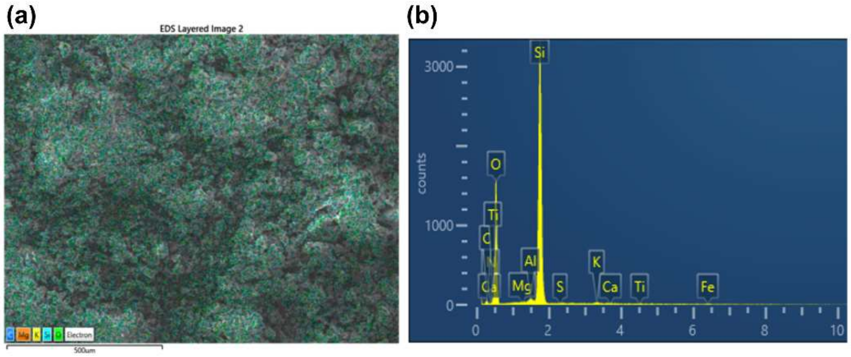

Figure S3. EDS layered image (a) and map spectrum (b) of leca sample after silica shell formation.

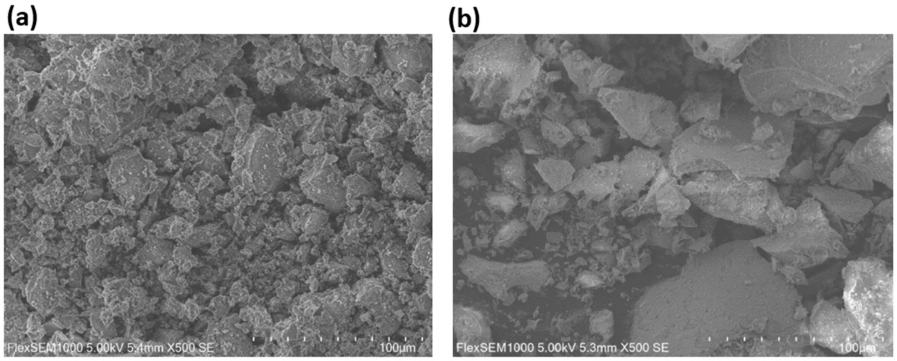

Figure S4. Leca samples before (a) and after (b) silica shell formation

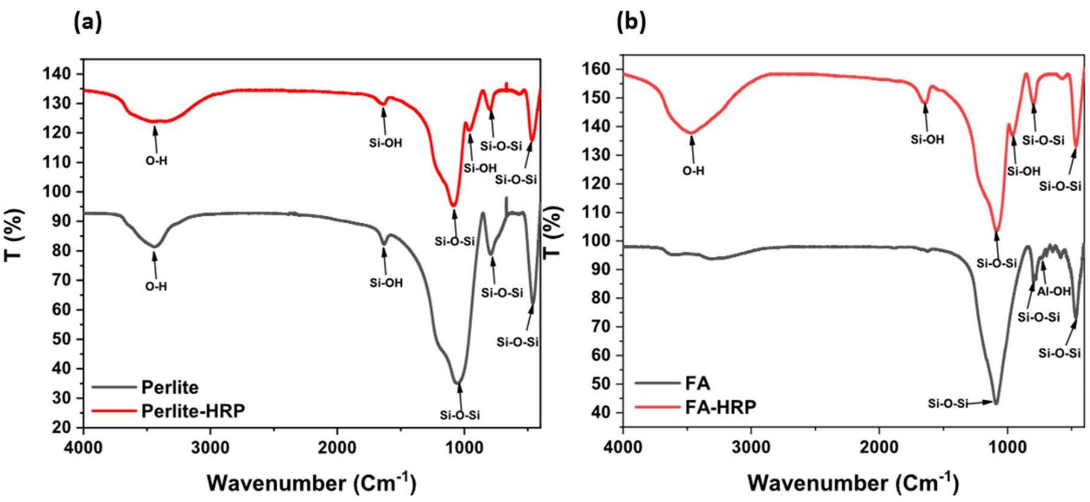

Figure S5. FTIR spectra of bare perlite (a) and FA (b) compared to core-shell immobilized perlite and FA.

Table S3. The difference of enzyme loading measured by Bradford assay and enzyme activity test

Biocatalyst	Enzyme activity test	Bradford assay
HRP-leca	7.02 U/mg	7.1 U/mg
HRP-perlite	7.5 U/mg	7.6 U/mg
HRP-FA	6.4 U/mg	6.4 U/mg
Laccase-Leca	0.038 U/mg	0.039 U/mg
Laccase-perlite	0.041 U/mg	0.041 U/mg
Laccase-FA	0.043 U/mg	0.045 U/mg

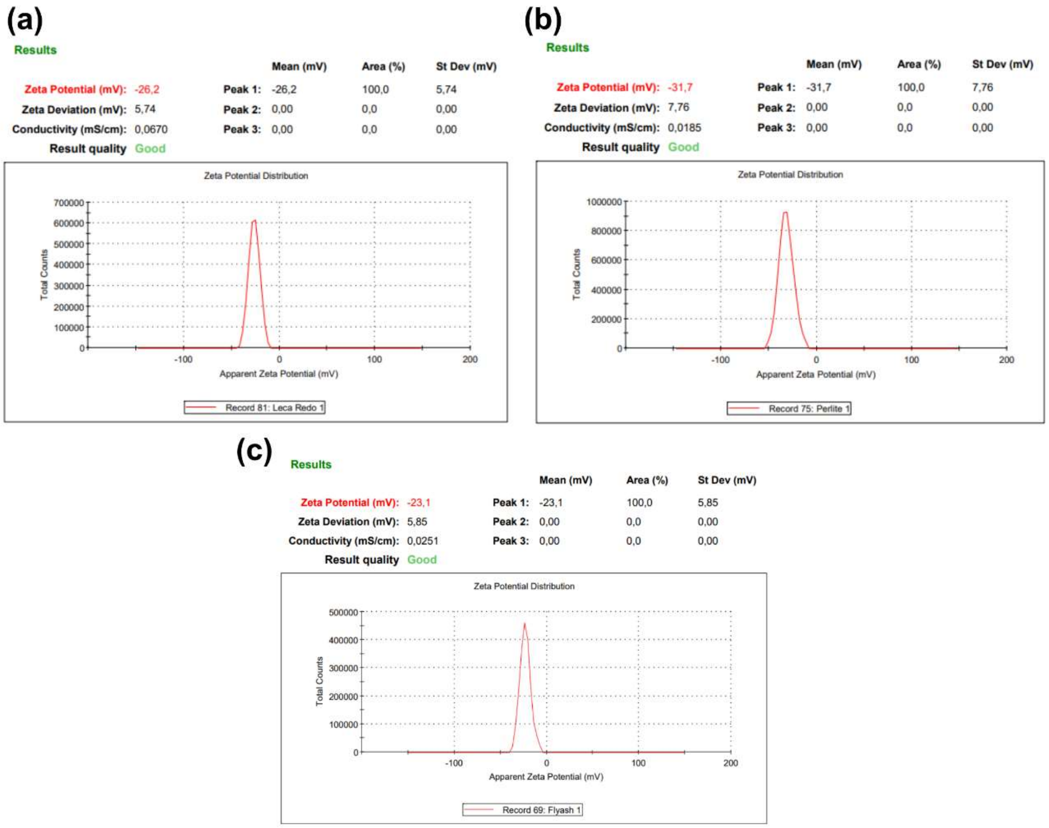

Figure S6. Zeta potential of natural silicates at natural pH: a) Leca, b) perlite, c) Fly ash

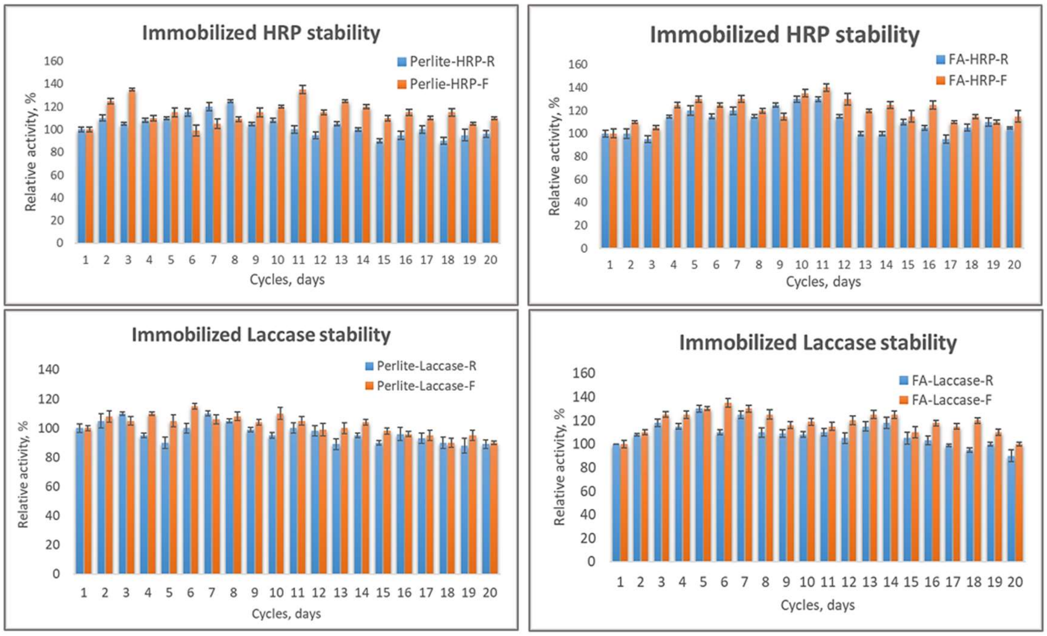

Figure S7. Immobilized HRP and laccase storage stability at room (R) and 4ºC temperature(F).

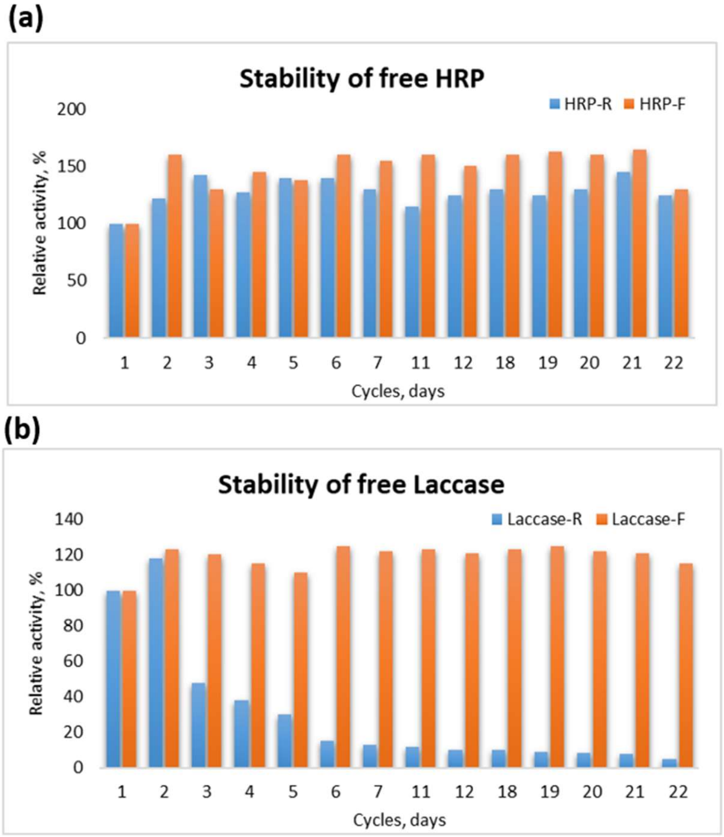

Figure S8. Storage stability of free enzymes at room (R) and 4 ºC temperature (F), with initial enzyme concentrations of 100 U/mL for HRP (a) and 20 U/mL for Laccase (b) in Potassium phosphate buffer, pH=6.

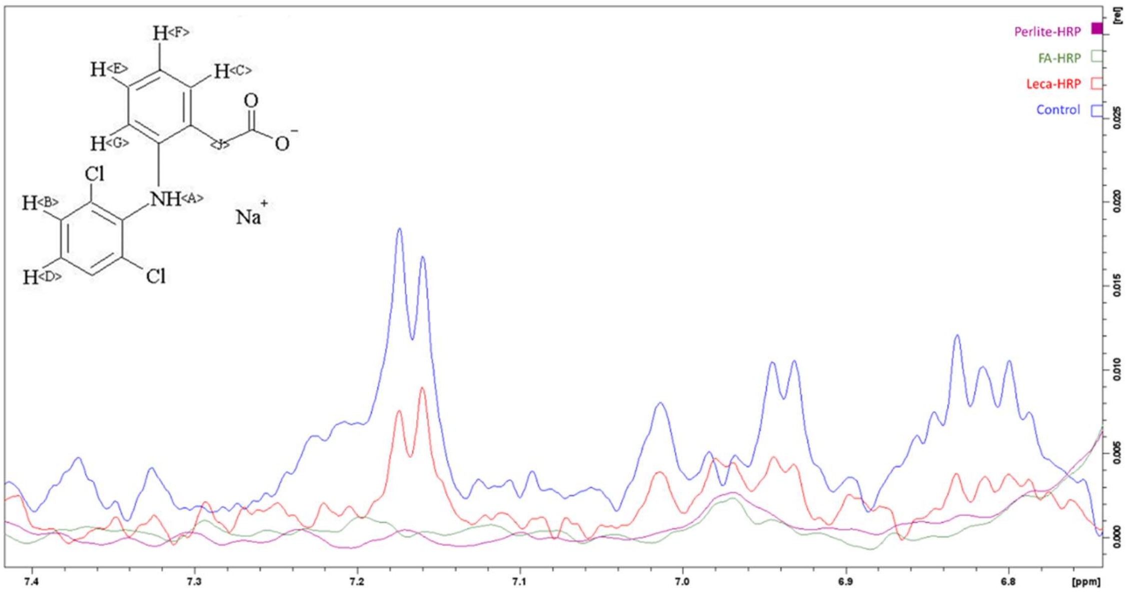

Figure S9. NMR spectra of DFC before and after interaction with immobilized HRP. The NMR sample solution was H_2O:D_2O 90%:10%.

**Assign.**	**Shift (ppm)**
B	7.38
D	7.08
E	6.93
F	6.8

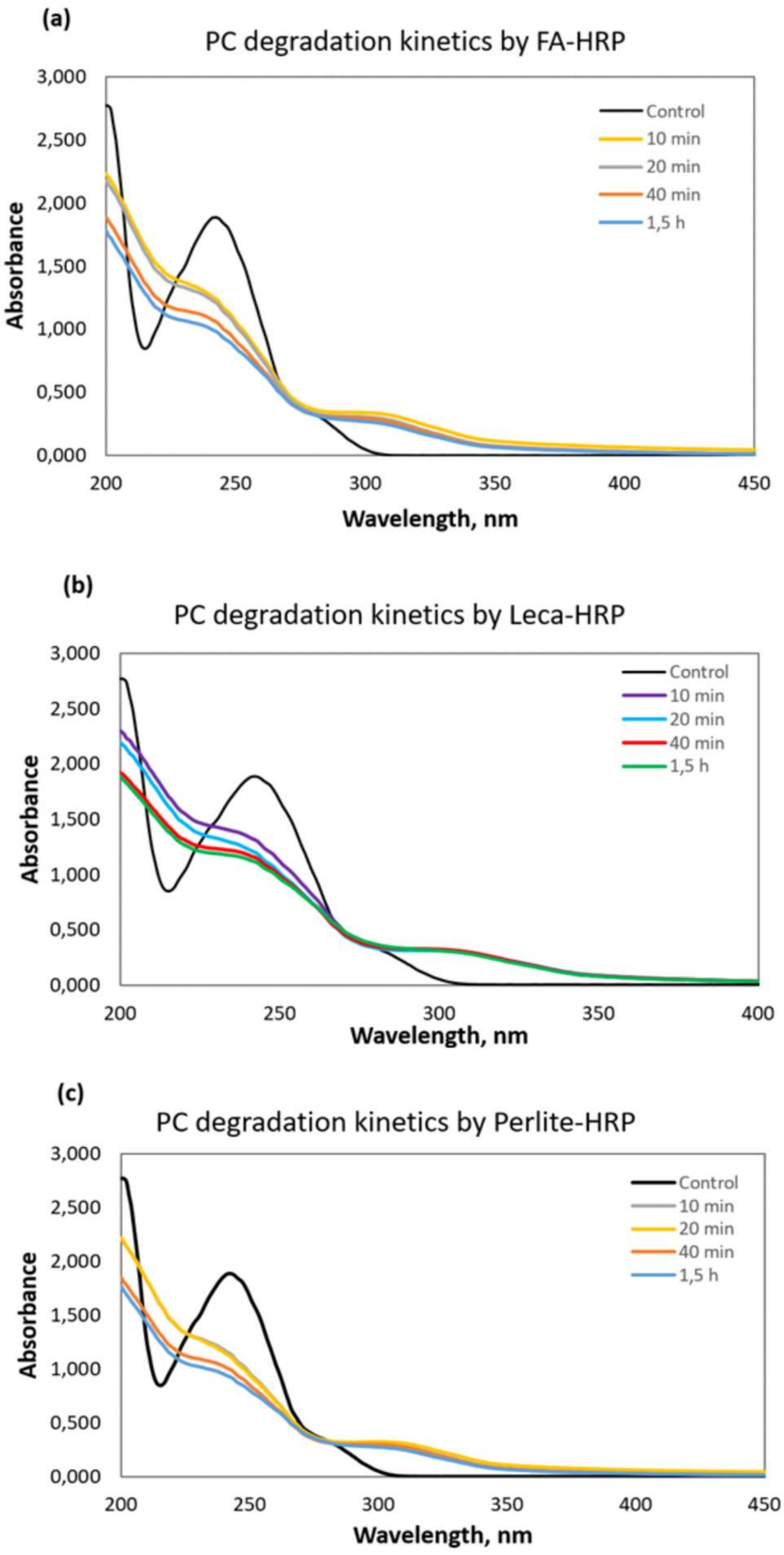

Figure S10. Paracetamol degradation kinetics by FA-HRP (a), Leca-HRP (b), and Perlite-HRP (c) samples (Initial PC concentration of 20 μg/mL, room temperature, pH=6.5, 3% H_2O_2).

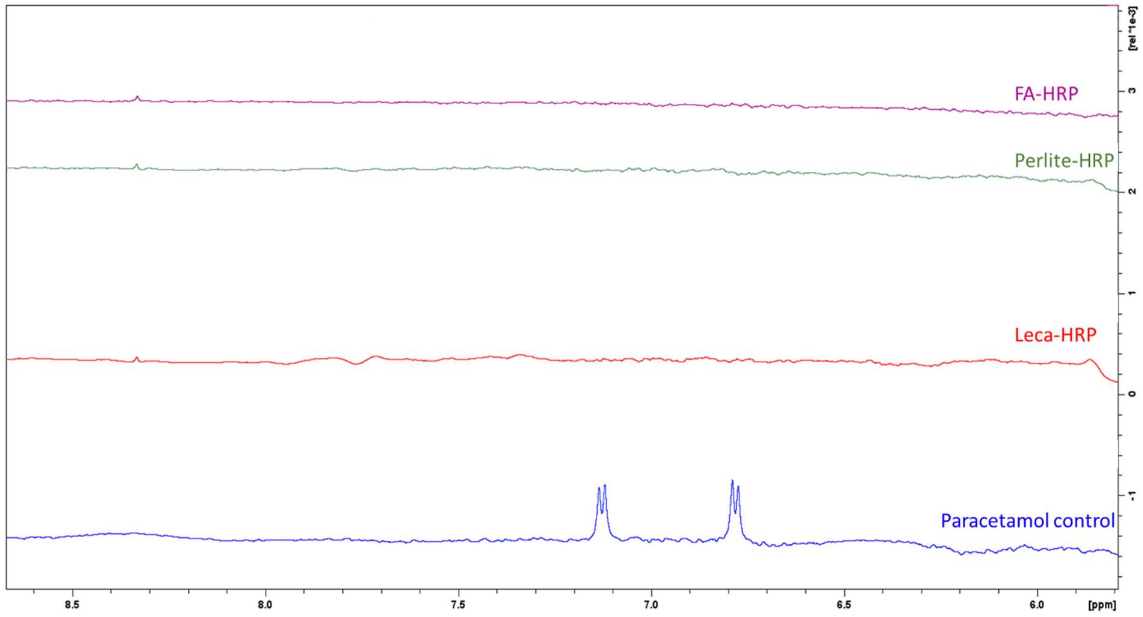

Figure S11. NMR spectra of initial Paracetamol (blue) and Paracetamol after interaction with sol–gel encapsulated Perlite-HRP, FA-HRP and Leca-HRP. The NMR sample solution was H_2O:D_2O 90%:10%.

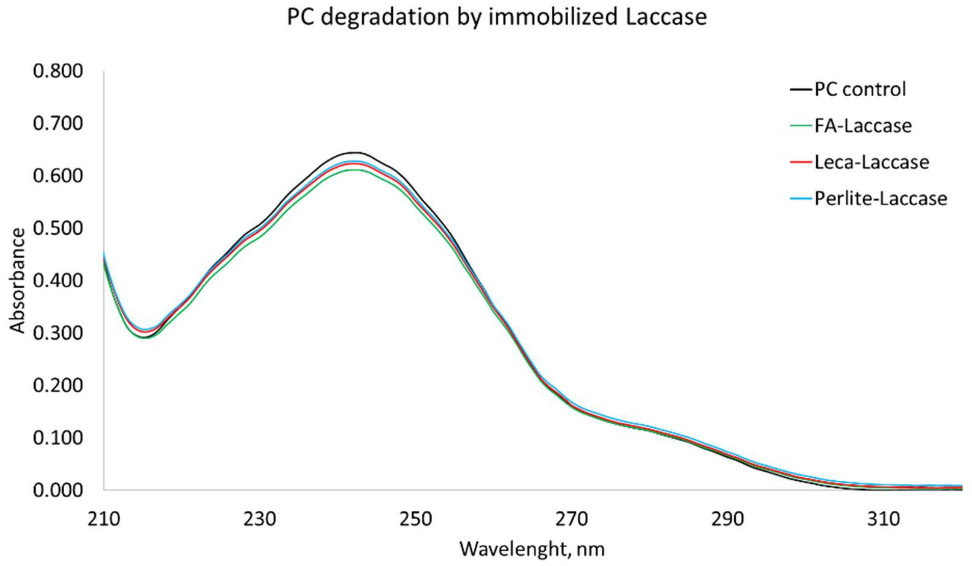

Figure S12. Paracetamol degradation by immobilized Laccase after 10 minutes interaction with the biocatalysts (Initial PC concentration of 10 μg/mL, room temperature, pH=6.5, 3% H_2O_2).

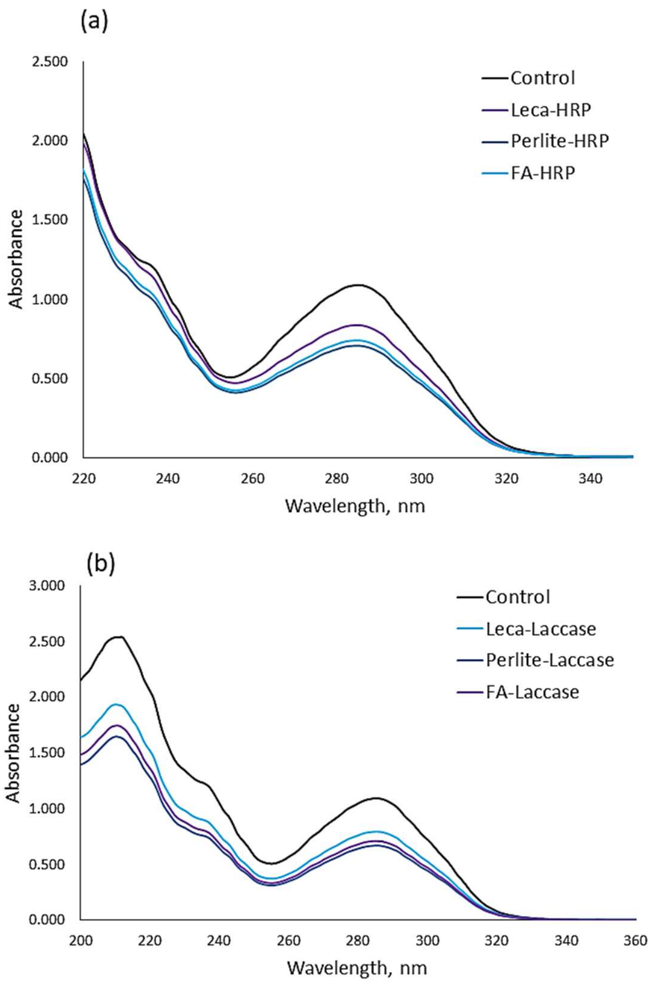

Figure S13. Carbamazepine degradation by immobilized HRP and Laccase samples measured by UV-VIS.

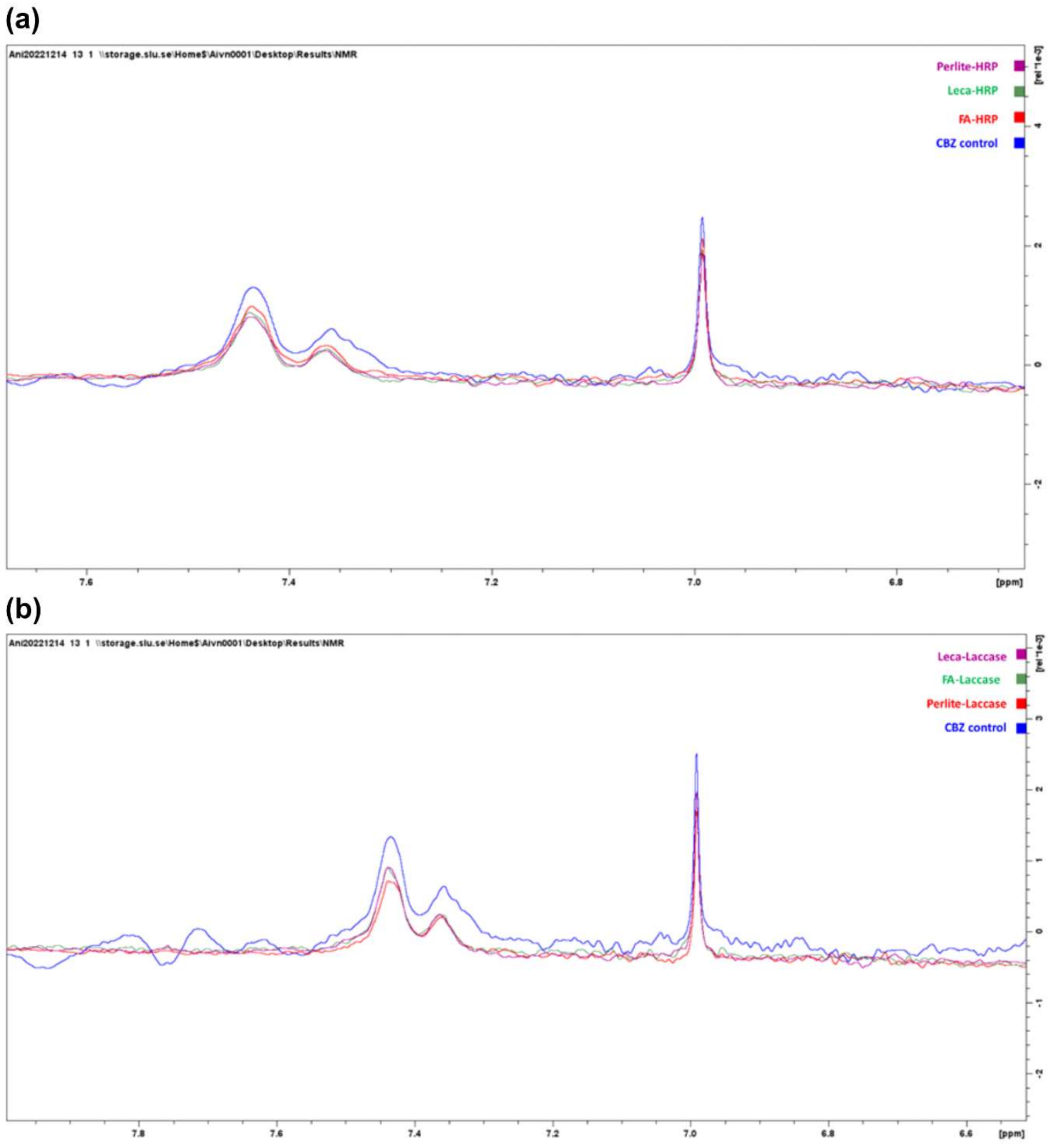

Figure S14. NMR spectra of initial Carbamazepine (blue) and Carbamazepine after interaction with sol–gel encapsulated Perlite-Laccase, FA-Laccase and Leca-Laccase (A) and HRP (B). The NMR sample solution was H_2O:D_2O 90%:10%.

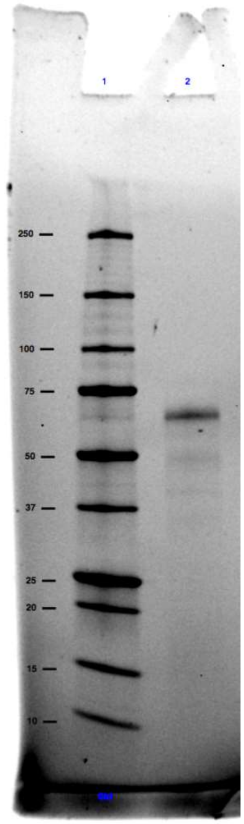

Figure S15. SDS-PAGE of Laccase enzyme.

Picnometric pore volume estimation

The densities of all three silicates were estimated by immersing 250 mg of samples in toluene in 5 ml picnometer and measuring the total weight for calculation of porosity, applying bulk aluminosilicate density for solid materials. Porosity was calculated from the differences in weights of estimated volumes of toluene.
